# Difference of Microbial Community in the Stream Adjacent to the Mixed Antibiotic Effluent Source

**DOI:** 10.3390/toxics12020135

**Published:** 2024-02-07

**Authors:** Jin-Wook Kim, Young-Kyu Hong, Oh-Kyung Kwon, Sung-Chul Kim

**Affiliations:** 1 Department of Bio-Environmental Chemistry, Chungnam National University, Daejeon 34134, Republic of Korea; kin1888@cnu.ac.kr (J.-W.K.); hyk8895@cnu.ac.kr (Y.-K.H.); 2 Biogas Research Center, Hankyung National University, Anseong 17579, Republic of Korea; okkwon1116@gmail.com

**Keywords:** livestock facilities, stream, gut bacteria, indicator

## Abstract

Released antibiotics from source to stream can influence bacterial communities and potentially alter the ecosystem. This research provides a comprehensive examination of the sources, distribution, and bacterial community dynamics associated with varied antibiotic release sources adjacent to the stream. The residual of antibiotics from different sources was determined, and the bacterial community structure was examined to reveal the differences in the bacteria community in the stream. The residual of antibiotics was quantified with liquid chromatography–tandem mass spectrometry (LC-MS/MS), and the Illumina MiSeq platform was utilized to sequence bacterial 16S rRNA genes, providing comprehensive insights into the bacterial community structure in the sediment across five different sites. Results indicated that the presence and distribution of antibiotics were significantly influenced by released sources. In the case of the bacterial community, the Proteobacteria and Firmicutes were the most dominant phyla in the sediment, and especially, the Firmicutes showed higher abundance in sites mostly affected by livestock sources. Additionally, livestock gut bacteria such as *Clostridium saudiense*, *Proteiniclasticum ruminis*, and *Turicibacter sanguinis* were prevalent in antibiotic-contaminated sites adjacent to livestock facilities. Overall, this study provides critical insights into the effect of antibiotic contamination by verifying the relationship between the occurrence of antibiotic residuals and the alteration in the bacterial community in the stream.

## 1. Introduction

Released antibiotics in the environment have raised concerns because of their adverse effects on human health and the alternation in the bacterial community in the ecosystem [1,2,3]. Extensive use of antibiotics in humans or livestock for therapeutic, prophylactic, and growth-promotion applications has been observed due to global population growth and rising demand for livestock products [4,5]. Consequently, unmetabolized forms of antibiotics can be released into the environment via discharged wastewater, animal manure compost applied in the agricultural field, and accidental leakage of animal manure in storage [6,7].

The sedimentary environment is a critical point in ecosystems. Due to its role in the geologic and hydrologic cycles, it acts as a significant circulation point for antibiotics in the ecosystem [8,9]. Particularly in areas with intensive livestock farming where antibiotics are routinely used, sediment in the watershed becomes a junction where both natural processes and anthropogenic activities converge, leading to a significant accumulation of these compounds [10,11,12]. In addition, the sediment ecosystem serves as a biodiverse habitat for innumerable bacterial communities, contributing significantly to maintaining ecological equilibrium through processes such as the cycling of nutrients and the degradation of complex organic matter [13,14,15]. These communities, despite forming intricate networks of interaction and interdependence that are typically robust under normal conditions, are highly sensitive to changes in the physical and chemical properties of the aquatic environment [16,17]. The introduction of antibiotics into the sedimentary environment can instigate considerable disturbances, leading to significant structural and functional transformations within bacterial communities [18,19]. Such changes can potentially incite the emergence and spread of antibiotic-resistant bacterial strains [20,21]. Therefore, it is essential to study bacterial communities in the sedimentary environment to evaluate the effect of antibiotics on sediment ecosystems and to identify potential sources of contamination.

In recent years, there have been many studies that attempt to understand the relationship between antibiotic contamination and bacterial community structure in sedimentary environments [22,23,24]. Notably, these investigations have increasingly utilized culture-independent approaches, focusing specifically on the use of 16S rRNA gene sequencing. This technique, widely accepted as a powerful tool for bacterial community analysis, offers a comprehensive view of bacterial diversity by sidestepping the limitations of traditional culture-based methods [25,26]. The application of such molecular-based techniques has allowed us to reveal the relationships between antibiotic contamination and bacterial community structures in the environment [27,28]. However, despite the growing understanding of the impacts of antibiotics on bacterial communities, a significant gap in our knowledge regarding the relationship between antibiotic contamination sources and bacterial communities within sedimentary environments remains.

In response to this challenge, we propose an approach that hinges on the idea that gut bacteria from livestock could potentially serve as bioindicators of antibiotic contaminants in sediment. As antibiotics are excreted into the environment through livestock-related facilities, the accompanying gut bacteria are also likely to enter ecosystems, possibly influencing the indigenous bacterial communities [29,30]. Given that livestock manure is an important contributor to the transmission of antibiotics into the environment, the presence of enteric bacteria could potentially reflect the type and source of antibiotic contamination in the environment.

Our primary objective in this study is to examine the bacterial community in sediment samples collected from five distinct sites adjacent to the stream and located at different antibiotic release sources. Specifically, we focus on livestock gut bacteria that are possibly associated with residual antibiotics related to livestock. By conducting a thorough investigation of the spatial distribution of these bacterial communities and their association with antibiotic contamination, we could identify the potential bacterial indicators of antibiotic contamination and verify the impact of livestock-related antibiotics in the stream.

## 2. Materials and Methods

### 2.1. Chemicals and Reagents

The target antibiotics were selected based on their sales in South Korea. A total of 10 antibiotics belonging to four groups were analyzed: Penicillins, including ampicillin (AMP) and penicillin G (PNG); tetracyclines, including chlortetracycline (CTC), oxytetracycline (OTC), and tetracycline (TC); macrolides, including tylosin (TYL); sulfonamides, including sulfadiazine (SDZ), sulfamethazine (SMZ), sulfamethoxazole (SMX), and sulfathiazole (STZ). The standard materials for the target analytes were acquired from Sigma-Aldrich (St. Louis, MO, USA). Simeton was used as an internal standard and purchased from Sigma-Aldrich (St. Louis, MO, USA). High-performance liquid chromatography (HPLC)-grade methanol, acetonitrile, and water were obtained from J. T. Baker (Phillipsburg, NJ, USA). All other chemicals used in this study were of analytical grade.

### 2.2. Site Description and Sample Collection

**Figure 1:**
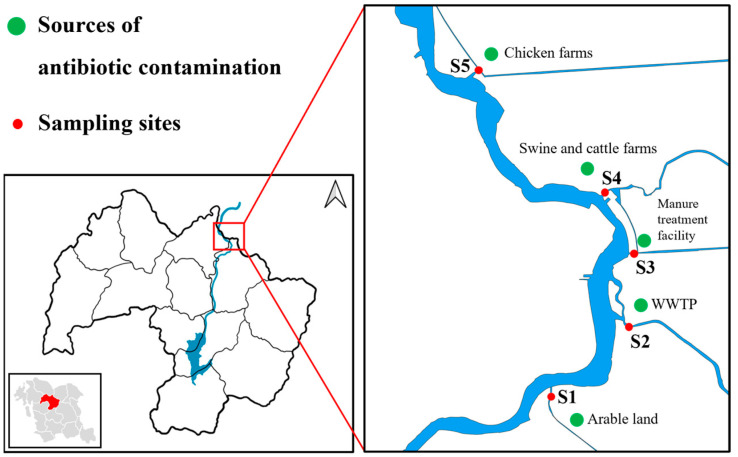
Map of sampling sites and sources of antibiotics contamination.

This study was conducted in the Muhan stream, located in Chungnam Province, South Korea (Figure 1). Surface water and sediment samples were collected from five sampling sites associated with different potential sources of antibiotic contamination. Site 1 is surrounded by arable land-applied animal manure compost, hence posing the risk for residual antibiotics to be transported from the soil into the surface water via rainfall. The wastewater treatment plant (WWTP) and the livestock manure treatment facility (LMTF) are located at sites 2 and 3, respectively, and the effluent from these facilities is discharged into the stream. The annual average streamflow of mainstream in August was 55.2 m^3^ min^−1^ and the average effluent flow of WWTP and LMTF was 15.3 and 0.1 m^3^ min^−1^ respectively. Operation of WWTP was followed by conventional processes (screening, sedimentation, coagulation, and disinfection), and agricultural fields, including greenhouses, were located near the WWTP and LMTF. Site 4 is adjacent to an intensive livestock farming area that comprises one beef and six swine farms, together holding a total of approximately 300 beef cattle and 18,000 swine. Site 5 is adjacent to poultry farms housing approximately 350,000-layer chickens. All livestock farms have waste storage containing manure and urine. The presence of these livestock farms suggests a risk of releasing residual antibiotics because of leakage in storage or inappropriate management of animal waste.

Surface water samples were collected in a 1.0 L brown glass bottle and stored at 4 °C until analysis. Sediment samples were collected using a stainless-steel shovel or hand auger. Approximately 200 g of sediment was collected from five randomly selected points, and the individual samples were combined into a single polyethylene bag to make one representative sample for each sampling site. Sediment samples were stored at 4 °C until analysis, and sub-samples were stored at −80 °C for subsequent microbiome analysis.

### 2.3. Physicochemical Analysis for Surface Water and Sediment

For surface water samples, water pH and electrical conductivity (EC) were determined using a pH meter (Orion Star™ A111, Thermo Fisher Scientific, Waltham, MA, USA) and an EC meter (SevenCompact™ Conductivity Meter S230, Mettler Toledo, Columbus, OH, USA), respectively. Total organic carbon (TOC) was measured using a TOC analyzer (Vario TOC cube, Elementar, Langenselbold, Germany). For total nitrogen (T-N) and total phosphorus (T-P) concentrations, surface water samples were digested using either an alkaline potassium persulfate (for T-N) or a potassium persulfate solution (for T-P) at 120 °C for 30 min, followed by quantification using a UV spectrophotometer (UVmini-1240, Shimadzu, Kyoto, Japan) by wavelengths of 220 nm (for T-N) and 880 nm (for T-P).

The sediment samples were oven-dried (JSOF-150, JSR, Tokyo, Japan) at 105 °C for 24 h for physicochemical analyses. Sediment texture was classified using the hydrometer method based on the United States Department of Agriculture’s (USDA) soil textural triangle. The sediment pH and EC were determined using a pH meter and an EC meter on a 1:5 (*w v*^−1^). Sediment organic matter (SOM) content was determined by the Walkley–Black method [31] and quantified at a wavelength of 610 nm using a UV spectrophotometer (UVmini-1240, Shimadzu, Kyoto, Japan). Total carbon (TC) and total nitrogen (TN) concentrations were analyzed using an elemental analyzer (TrueSpec CHN, LECO Corporation, St. Joseph, MI, USA).

### 2.4. Antibiotics Analysis

#### 2.4.1. Antibiotics Extraction and Clean-Up Process

Prior to analysis, surface water samples were filtered through a 0.45 μm cellulose acetate membrane filter, and sediment samples were air-dried at 20 °C under dark conditions. The extraction of antibiotics from the surface water and sediment samples was conducted using a solid-phase extraction (SPE) method, as previously described [32]. In brief, the extraction and clean-up processes are as follows: The pH of surface water samples (120 mL) was adjusted to 2.5 with 40% H_2_SO_4_ (*v v*^−1^), added 500 μL of 5% Na_2_-EDTA, and shaken for 15 min on the orbital shaker. Sediment samples (1.0 g) were extracted with 20 mL of McIlvain buffer (pH 4.0) and 250 μL of 5% Na_2_-EDTA, then they were shaken for 15 min and centrifuged at 4000 rpm for 15 min. The supernatant was transferred into 250 mL Erlenmeyer flasks, and these procedures were repeated two times (total of 40 mL). The combined supernatant was diluted to 120 mL with ultrapure water and filtered using a 0.22 μm cellulose acetate membrane filter.

Pretreated samples were purified by an Oasis HLB Extraction Cartridge (3 cc/60 mg, Water, Milford, MA, USA) with a Visiprep SPE vacuum manifold (Supelco, Bellefonte, PA, USA), and the analytes were eluted using 5 mL of methanol. Finally, the extracts were concentrated using a nitrogen evaporator (12 Position N-EVAP Nitrogen Evaporator, Organization, Berlin, MA, USA), reconstituted with 120 μL of 0.1% formic acid in HPLC water, and filtered using a 1.5 mL centrifuge tube (Spin-X centrifuge tube filter, Corning Incorporated, Corning, NY, USA) containing a 0.22 μm nylon filter, and then centrifuged at 15,000 rpm for 3 min (Figure S1).

#### 2.4.2. Instrumental Analysis

High-performance liquid chromatography (HPLC, 1290 Infinity II, Agilent, Santa Clara, CA, USA) coupled with triple quadrupole mass spectrometry (6500 Qtrap, SCIEX, Framingham, MA, USA) equipped with an electrospray ionization probe was used for the detection and quantification of antibiotic residuals in samples. All target analytes were analyzed using multiple reaction monitoring (MRM) in the positive ion mode. The chromatographic separation was performed using a Zorbax Eclipse Plus-C18 column (Agilent, 4.6 × 150 mm, 3.5 μm). The gradient elution system consisted of a flow rate of 0.7 mL min^−1^ with mobile phases A: HPLC-grade water with 0.1% formic acid and B: acetonitrile in 0.1% formic acid. The detailed HPLC and mass spectrometry conditions are presented in Tables S1 and S2.

#### 2.4.3. Method Validation

The analytical procedure employed in this study was validated in terms of specificity, linearity, matrix effect, accuracy, precision, and method detection limit (MDL) and limit of quantification (LOQ) (Tables S3–S6, Figures S2 and S3), as previously documented [32]. Briefly, the method exhibited high specificity and superior linearity, accurately quantifying target analytes without interference across a broad concentration range. The MDL and LOQ values for each analyte ranged from 1.2 to 13.2 ng kg^−1^ and 4.0 to 42.0 ng kg^−1^, respectively, which confirms the adequacy of this method for the quantification of trace amounts of veterinary antibiotics (VAs) in the environment. Accuracy, represented as a percentage of recovery at three concentration levels, indicated that most VAs exceeded 70%; for those with lower recoveries, the internal standard method was employed to enhance quantification.

### 2.5. Microbiome Analysis

#### 2.5.1. DNA Extraction and 16S rRNA Gene Amplicon Sequencing

For the microbiome analysis, sediment samples were freeze-dried using a freeze-dryer (SFDSF12, SAMWON, Seoul, Republic of Korea). Total DNA was extracted using a commercial DNA extraction kit (NucleoSpin^®^ Soil, Macherey-Nagel, Düren, Germany). Briefly, 0.3 g of the sediment sample was placed in MN Bead Tubes Type A, which contained ceramic beads. Then, 700 μL of lysis buffer (SL1) and 150 μL of enhancer SX were added, followed by a bead-beating procedure using a Mini Beadbeater-16 (BioSpec Products, Bartlesville, OK, USA). The subsequent procedures were carried out according to the manufacturer’s protocol, with the total DNA being extracted with 50 μL of elution buffer SE. Finally, the concentrations and purities of the extracted DNA were evaluated using a UV-Vis spectrophotometer (Optizen NANO Q, Mecasys, Daejeon, Republic of Korea).

Total DNA samples were sent to LabGenomics (Seongnam, Republic of Korea) for microbiome analysis, which involved PCR amplification and Illumina MiSeq sequencing. Universal primers 341F (5′-CCTACGGNGGCWGCAG-3′) and 805R (5′-GACTACHVGGGGTATCC-3′) were used to amplify the V3–V4 regions of the 16S rRNA gene for sediment bacterial community structure analysis. PCR was performed using Herculase II Fusion DNA Polymerase (Agilent, Santa Clara, CA, USA), and PCR mixtures comprised a total volume of 23 μL, which included 2.5 μL of amplicon PCR F, R primer, 0.5 μL of Herculase ΙΙ Fusion DNA polymerase, 5.0 μL of 5 × Herculase ΙΙ reaction buffer, 0.25 μL of dNTPs (100 nM), and 14.75 μL of PCR-grade water. The amplicon PCR conditions involved an initial denaturation step at 95 °C for 3 min, followed by 25 cycles of denaturation (95 °C for 30 s), annealing (55 °C for 30 s), and extension (72 °C for 30 s), with a final extension at 72 °C for 5 min and then held at 4 °C. Following this, limited-cycle amplification was performed to attach multiplexing indices and Illumina adapter sequences, and the conditions were as follows: An initial denaturation step at 95 °C for 3 min, followed by eight cycles of denaturation (95 °C for 30 s), annealing (55 °C for 30 s), and extension (72 °C for 30 s), with a final extension at 72 °C for 5 min and then held at 4 °C. The final product was normalized and pooled using PicoGreen (Promega, Madison, WI, USA), and the library size was verified using the TapeStation DNA screentape D1000 (Agilent). Finally, paired-end sequencing was conducted using the MiSeq™ platform (Illumina, San Diego, CA, USA).

#### 2.5.2. Bioinformatic Analysis

The raw sequence data obtained from Illumina MiSeq sequencing analysis were sorted per sample via the index sequence, and the FASTQ files were generated. Then, individual samples’ paired-end data were assembled into a single sequence using FLASH (version 1.2.11) [33], and sequences shorter than 400 bp or exceeding 500 bp were discarded. The CD-HIT-OTU software was used to process the retrieved sequences to filter out low-quality, ambiguous, and chimeric sequences, which are seen as sequencing errors. The refined sequences were then clustered into species-level operational taxonomic units (OTUs) with 97% sequence similarity. For taxonomic assignment, the representative sequence from each OTU was compared to the reference sequence database (NCBI 16S Microbial) using BLAST+ (version 2.9.0) [34]. Further bioinformatics analyses were conducted using QIIME (version 1.9) with the obtained OTU data and taxonomic information [35]. To evaluate the alpha diversity in the bacterial community in five sediment samples, both the Chao 1 and Shannon indices were calculated. Additionally, the beta diversity was assessed based on the weighted UniFrac distance [36], and principal coordinate analysis (PCoA) was utilized to compare and illustrate the diversity of bacterial communities across the five sediment groups.

### 2.6. Statistical Analysis

Statistical analyses were performed using the Statistical Package for Social Science (SPSS) version 26.0 (2021, SPSS Inc., Chicago, IL, USA). The physicochemical properties and antibiotic concentration of surface water and sediment were each measured in triplicate and expressed as mean and standard deviation. The one-way analysis of variance (ANOVA) was performed to verify the statistical differences among the physicochemical results, followed by the post-hoc Duncan’s test (*p* < 0.05) for multiple comparisons between sample groups. Cluster analysis was conducted with hierarchy cluster analysis, adapting the Ward method. 

## 3. Results

### 3.1. Physicochemical Properties of Surface Water and Sediment

The physicochemical properties of the surface water and sediment samples are summarized in Table 1 and Table 2. For surface water samples, pH and EC values ranged from 6.98 to 7.87 and 0.28 to 18.69 dS m^−1^, respectively. The mean concentration of TOC, T-N, and T-P contents ranged from 5.81 to 265.96 mg L^−1^, 4.11 to 289.43 mg L^−1^, and 0.24 to 9.77 mg L^−1^, respectively. For the sediment samples, the pH and EC values ranged from 6.87 to 7.71 and 0.39 to 16.23 dS m^−1^, respectively. The mean concentrations of SOM, T-C, and T-N contents ranged from 0.91 to 3.36%, 2.87 to 19.10 g kg^−1^, and 0.99 to 4.86 g kg^−1^, respectively. The highest EC, TOC, T-N, and T-P in both surface water and sediment were observed at site 3, where a livestock manure treatment facility was nearby.

**Figure 2:**
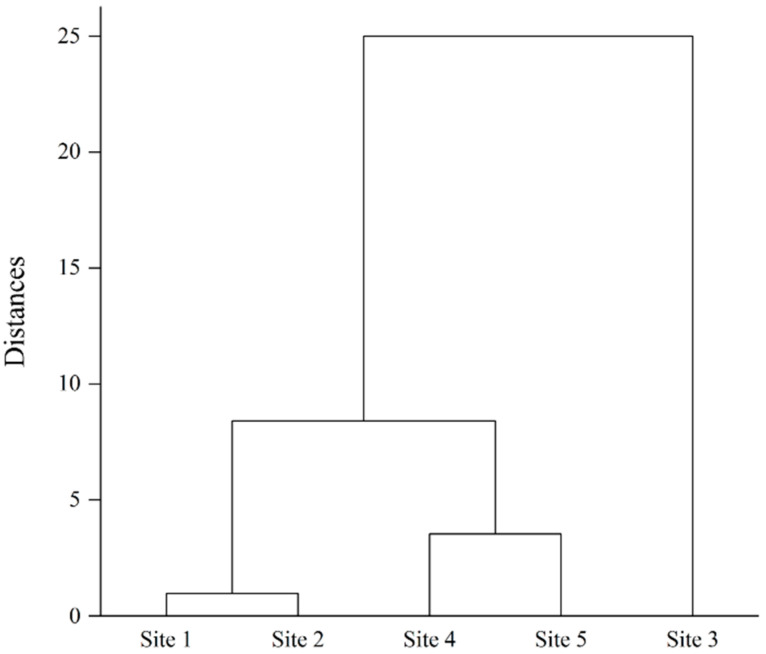
Cluster analysis of sampling sites based on chemical properties and concentration of antibiotics in surface water and sediment.

The chemical properties and detected antibiotic concentrations of surface water and sediment were used for cluster analysis (Figure 2). Each sampling site was clearly divided into 3 groups (sites 1 and 2, sites 4 and 5, and site 3). The effluent of livestock wastewater was directly released from the facilities at site 3, and condensed livestock feeding operations of swine, poultry, and cows were located at sites 4 and 5. Thus, sampling sites 3, 4, and 5 were considered to be livestock-related sites. The agricultural field and wastewater treatment plant were located at sites 1 and 2, considering the human-impacted area. This result indicates that the characteristics of the antibiotic release source are different and may have different effects on the bacterial community in the stream.

### 3.2. Antibiotics Concentrations of Surface Water and Sediment

The concentrations of the target antibiotics in surface water and sediment collected from the study area are presented in Table 3. Seven of the ten antibiotics were detected in surface water and sediment samples, with concentrations ranging from 0.01 to 0.25 μg L^−1^ and 1.45 to 9.04 μg kg^−1^, respectively. While the detection frequency of antibiotics in sediment samples was lower compared to surface water samples, their higher concentrations reflect an accumulation of antibiotics released from antibiotic contamination sources. Tetracycline and sulfonamides were the most frequently detected classes of antibiotics, while macrolides were rarely detected, and penicillins were not found in any of the samples. Sulfamethazine was particularly prevalent in sediments, with concentrations ranging from 3.70 to 4.96 μg kg^−1^, and the tetracycline group (chlortetracycline, oxytetracycline, and tetracycline) was most frequently detected in surface water, with concentrations from 0.08 to 0.31 μg L^−1^. The residuals of antibiotics showed varying distributions across sites. At site 1, which is close to agricultural drainage ditches, the greatest diversity of antibiotics was found in surface water samples. Additionally, sites 3, 4, and 5, which are adjacent to livestock farms and manure treatment facilities, also showed the presence of various antibiotics. However, at site 2, located near a wastewater treatment plant (WWTP), no antibiotics were detected in either the surface water or the sediment.

### 3.3. Diversity of Bacterial Communities in Sediments

Read counts, OTU number, Good’s coverage, and alpha diversity indices (Chao1 and Shannon) were obtained by high-throughput Illumina sequencing of the V3–V4 region of the 16S rRNA gene in this study (Table 4). A total of 583,133 raw read sequences were determined from the five sediment samples, and low-quality and chimeric sequences were removed through preprocessing and clustering using CD-HIT-OTU, obtaining a total range of 15,046 to 22,499 read counts. Subsequently, these filtered sequences were clustered into OTUs with a similarity level of 97%, resulting in a total range of 1476 to 1741 OTUs being identified. The Good’s coverage values range from 94.6% to 97.3%, indicating that the sequencing depth was sufficient to recover most of the bacterial communities in the sediment samples. The Chao1 index, a measure of species richness, ranged from 1903 to 2285, with the highest bacterial diversity observed at site 5 and the lowest at site 2. The Shannon index, commonly used to quantify species diversity, ranged from 8.51 at site 4 to 9.16 at site 1.

### 3.4. Bacterial Community Structure in Sediments

**Figure 3:**
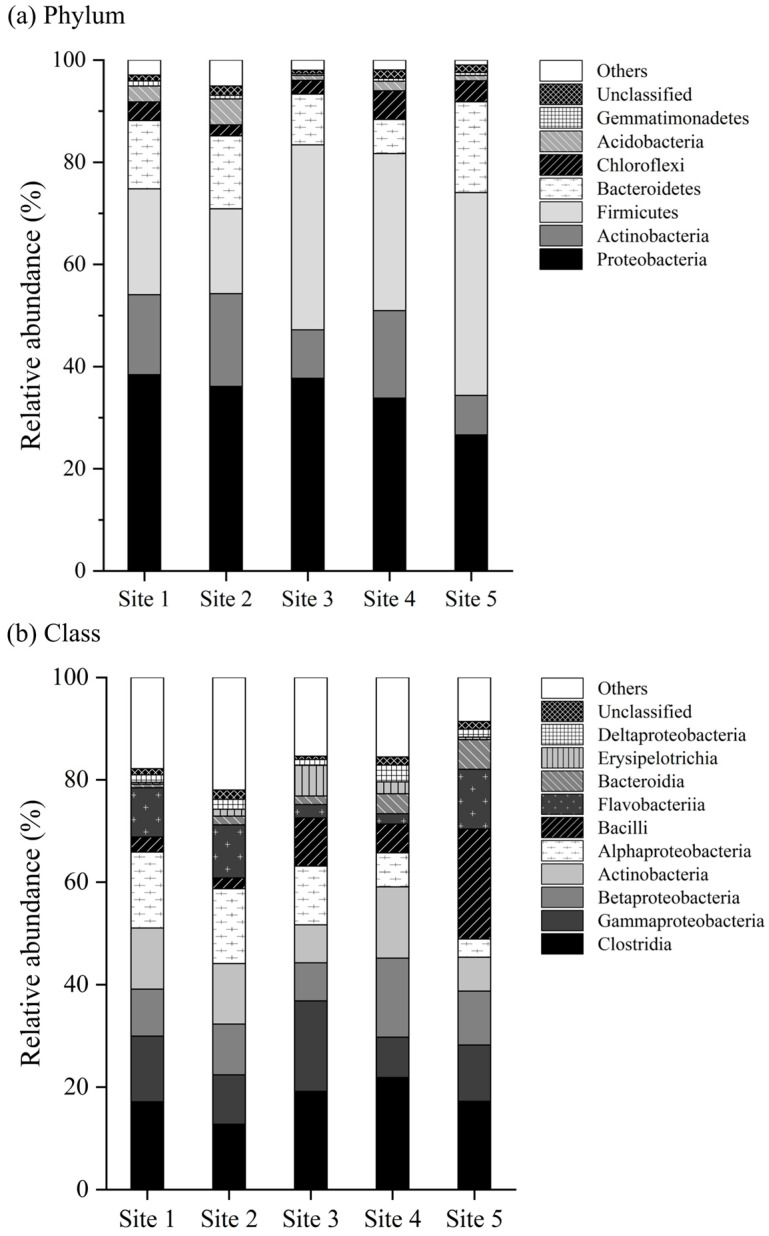
Relative abundance of sediment bacteria at the phylum and class level. All phyla and classes with an abundance below 1% (including the unclassified bacteria) are consolidated into “others”. (**a**) Phylum and (**b**) class level.

The bacterial community composition and structure in the sediment adjacent to different antibiotic release sources located along the stream were analyzed at the phylum and class levels (Figure 3). A total of 23 phyla, 58 classes, 130 orders, 274 families, 795 genera, and 1368 species were identified in the study area. At the phylum level, Proteobacteria was the predominant phylum in most samples, with its relative abundance ranging from a minimum of 26.6% at site 5 to a maximum of 38.4% at site 1 (Figure 3a). Four other phyla, including *Firmicutes* (16.6–39.7%), *Actinobacteria* (7.7–18.2%), *Bacteroidetes* (6.7–17.8%), and *Chloroflexi* (2.1–5.6%), also exhibited a higher relative abundance. (Figure 3a). Among these dominant bacteria, a comparison of the abundances across the five sites showed that the abundances of Firmicutes differed among varied antibiotic release sources. Firmicutes was the most abundant phylum at site 5, which is adjacent to a poultry farm, accounting for a relative abundance of 39.7%, and this was the second abundant phylum at sites 3 and 4, with relative abundances of 36.2% and 30.7%, respectively. However, at sites 1 and 2, the distribution of Firmicutes was relatively lower, with relative abundances of 20.7% and 16.6%, respectively.

At the class level, *Clostridia* was the predominant class in most samples (12.75–21.86%), followed by *Gammaproteobacteria* (7.87–17.65%), *Betaproteobacteria* (7.42–15.43%), *Actinobacteria* (6.61–13.92%), *Alphaproteobacteria* (3.58–14.83%), *Bacilli* (2.09–21.42%), and *Flavobacteriia* (2.04–11.69%), with their distributions showing differences across sampling sites (Figure 3b).

**Figure 4:**
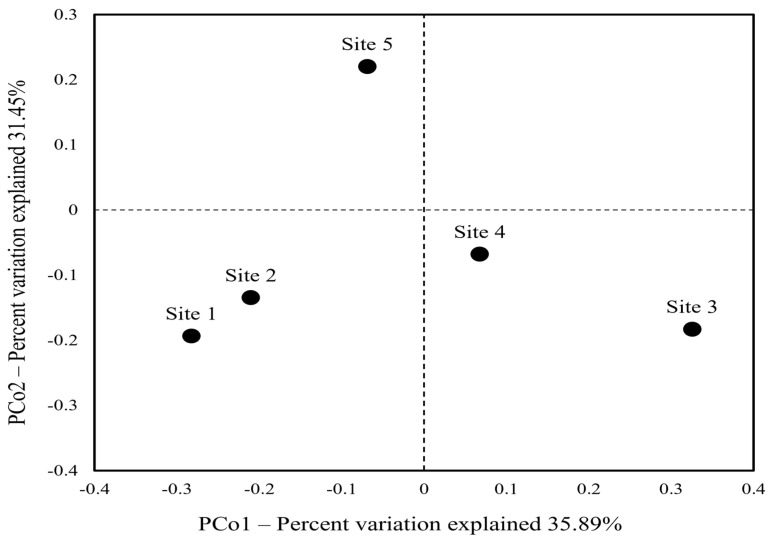
Principal coordinate analysis (PCoA) of bacterial communities based on Bray–Curtis dissimilarity.

The principal coordinate analysis (PCoA) with Bray–Curtis dissimilarity illustrates the similarity of the bacterial community composition across five sediment samples (Figure 4). The PCoA plot clearly separated sediment samples, which signifies that the bacterial community structure of sediment differs among sites and could be influenced by released antibiotics. More specifically, sites 1 and 2 are closely clustered together, suggesting a similar community composition in these locations. In contrast, the other samples (sites 3, 4, and 5) are separated from each other, indicating a difference in the microbial community compositions of these sediments.

### 3.5. Distribution of the Gut Bacteria in Sediment Samples

This study investigated the relationship between the concentration of antibiotic residuals and bacterial communities in sedimentary environments. Considering that antibiotics are typically released into the environment through livestock manure, it was anticipated that the bacterial community in antibiotic-contaminated sediments would include gut bacteria from livestock. Bacteria found in the gastrointestinal tract or feces of humans and livestock, as reported in previous studies, served as the reference for this investigation. Among these, human and livestock gut bacteria detected in sedimentary samples in this study with a relative abundance of 0.1% or higher are documented in Table 5.

Within these sediment samples, the detected intestinal bacteria predominantly belonged to the Firmicutes phylum, where the Clostridium genus emerged as the most prevalent. Species such as *Clostridium saudiense* and *Clostridium butyricum* were consistently found across all sites, demonstrating relative abundances of 0.07 to 8.10% and 0.26 to 3.16%, respectively. *Turicibacter sanguinis* and *Proteiniclasticum ruminis*, also from the Firmicutes phylum, presented high detection frequencies, with their abundances ranging between 0.23 and 5.97% and 0.24 and 4.34%, respectively. Bacterial species from other phyla, such as *Corynebacterium humireducens*, *Moheibacter stercoris*, and *Spaerobacter thermophilus*, were also identified, suggesting that sedimentary environments may harbor a diverse range of gut bacteria from both humans and livestock. Notably, the distribution of these gut bacteria varied by site, with the majority of these species detected, particularly at sites 3 and 4. These sites demonstrated higher total gut bacterial abundances of 22.29% and 16.71%, respectively, in comparison to the other sites. This section may be divided by subheadings. It should provide a concise and precise description of the experimental results, their interpretation, and the experimental conclusions that can be drawn.

## 4. Discussion

Antibiotics are commonly introduced into streams through effluent from wastewater treatment or runoff from arable soils when livestock manure-based compost and liquid manure are applied to soil [37,38]. This study aimed to investigate the relationship between residual antibiotics and bacterial communities in the stream, focusing on the different antibiotic contamination sources adjacent to the stream.

The concentration of antibiotic residuals in the sediment was found to be higher than that in the surface water samples. This difference in antibiotic concentrations between sediment and surface water could be attributable to the continuous accumulation of antibiotics. Despite the comparatively lower antibiotic concentration in the surface water, the long-term adsorption and subsequent accumulation of antibiotics in the sediment contribute to their overall environmental persistence [39]. Among the four different antibiotic classes, tetracycline and sulfonamide groups are the most highly detected antibiotics in the study area. In the agricultural environment, the differences in the distribution of these antibiotic classes across the sites could be attributed to their usage and specific physicochemical properties. For instance, chlortetracycline, as an antibiotic with a wide spectrum of antibacterial activity, is commonly employed to prevent infection and promote growth in livestock [40,41], and is also widely used in the breeding of swine and chicken in South Korea. This extensive application to livestock inherently raises the likelihood of environmental exposure. In addition, the physicochemical properties of chlortetracycline contribute to its environmental persistence. It has a high adsorption coefficient (K_d_), which facilitates complex formation with divalent metal ions in the soil, thereby sustaining its presence in both soil and sedimentary environments [42,43]. Owing to its extensive use in livestock farming and its tendency for absorption and retention in soil and sediment, chlortetracycline has emerged as one of the antibiotics frequently detected in agricultural environments [44,45].

Sulfonamide antibiotics, due to their high water solubility, exhibit high environmental mobility, which allows them to readily migrate from soil to the watershed, and they pose potential ecological risks in aquatic environments [46,47]. Furthermore, sulfamethazine emerged as the predominant antibiotic detected in our study, with its occurrence being widespread in the sediment samples. Its high detection frequency in sediment, as also reported in other studies [48,49], suggests its notable tendency to accumulate and persist in these environments.

Contrary to the frequent detection of tetracyclines and sulfonamides in our study, penicillins, despite their extensive sales and use in South Korea, were seldom detected. This pattern of infrequent detection or lower concentrations of penicillins in environmental matrices such as surface water, wastewater, and soil is consistent with previous studies [50,51]. This observation can be explained by the unique physicochemical properties of penicillins, particularly their β-lactam structure, which makes them prone to rapid environmental degradation. This degradation can occur either through enzymatic activity by β-lactamase, a common product of many bacteria, or through chemical hydrolysis, thereby significantly decreasing their environmental half-life [52,53,54].

The highest concentration of detected antibiotics was mostly observed at sites 3 and 4, and livestock manure treatment facilities and livestock farms are located near the sampling sites. This observation suggests that effluents from these facilities could serve as sources of antibiotic contamination. Specifically, site 3, which is influenced by effluents from the manure treatment facility, exhibited the highest EC value and nutrient content in both surface water and sediment samples (Table 1 and Table 2). Previous studies also highlighted the role of livestock facilities as a major source of antibiotic pollution in agricultural water systems, as well as the impact of manure compost application [55,56,57]. Contrastingly, at site 2, adjacent to a wastewater treatment plant (WWTP) primarily treating wastewater from human activities, no antibiotics were detected in the stream. This result indicates that the effluent from this facility does not contribute to antibiotic contamination in the study area.

In the case of site 5, located near the chicken farm, oxytetracycline, tylosin, and sulfamethazine were detected in the surface water, and only sulfamethazine was detected in the sediment. Among the three detected antibiotics in the stream at site 5, only sulfamethazine was observed both in the surface water and in the sediment. This finding implies that the sorption of sulfamethazine was highly related to organic matter contents. Total organic content (TOC) in the surface water at sampling site 5 was the second highest among sampling sites (Table 1 and Table 2) and this high TOC may affect the high affinity of antibiotic sorption in surface water [58]. A similar trend was observed at site 1, showing only sulfamethazine was detected in the sediment, although six out of seven measurable antibiotics were detected in surface water.

In order to assess different bacterial communities depending on varied antibiotic release sources, metagenomic analysis was conducted by leveraging the Illumina MiSeq sequencing platform to sequence bacterial 16S rRNA genes, a reliable and widely accepted method for characterizing bacterial community structures [59,60]. Environmental bacteria, as sensitive bioindicators of their habitat, have the intrinsic ability to reflect changes in their surroundings [61]. They can adapt to shifts in environmental conditions through alterations in community structure, diversity, and functional potential [62]. Consequently, the structure of these bacterial communities can exhibit significant variations, reflecting the complex and multifaceted interactions between these microorganisms and their environment in response to a range of environmental pressures, such as alterations in nutrient availability, exposure to contaminants, or shifts in other key physicochemical parameters [63,64,65].

In our examination of the bacterial community structure and composition in the sediment, we found intriguing patterns of diversity and distribution. The bacterial phylum Proteobacteria was predominant across the four sites, followed by Firmicutes, Actinobacteria, Bacteroidetes, and Chloroflexi. The phylum Proteobacteria is known to be abundant in a variety of environments [66,67,68]. In addition, many previous studies have reported the predominance of Proteobacteria in sedimentary environments [69,70,71].

Interestingly, we observed variances in the distribution of the Firmicutes phylum across the five study sites, especially in relation to different antibiotic release sources. Site 5, adjacent to a poultry farm, showed the highest relative abundance of Firmicutes at 39.7%, making it the most abundant phylum at that location, followed by sites 3 and 4, with relative abundances of 36.2% and 30.7%, respectively. Conversely, sites 1 and 2 demonstrated a lower distribution of Firmicutes, with relative abundances of 20.7% and 16.6%, respectively. Firmicutes, along with Bacteroidetes, are the two most dominant bacterial phyla in the intestinal microbiota of humans and many animals [72,73,74], and thus environments in which firmicutes were detected in abundance are likely to be affected by fecal contamination [75,76]. Similarly, the Clostridia class, which is commonly found in the intestinal microbiota of many mammals, including humans, exhibited the highest relative abundance at site 4 and the lowest at site 2. This suggests the possibility of fecal contamination influencing the bacterial community composition at these locations.

Building upon the observed relationships between site-specific bacterial communities and livestock related effluent sources, we examined livestock gut bacteria at species level to identify potential indicator organisms that could help more directly identify antibiotic contamination sources in each site. Most of the livestock intestinal bacteria identified in the sediment were members of the Firmicutes phylum, more specifically of the Clostridium genus, suggesting these species could serve as indicators of livestock-sourced fecal contamination. Among the diverse bacterial taxa detected across the five sedimentary environments, one notable finding was the relative abundance of *Clostridium saudiense* at sites 3 and 4. *C. saudiense* is primarily isolated from swine manure slurry, an observation corroborated by several recent studies [77,78,79]. Given the proximity of site 3 to a livestock manure treatment facility and the location of site 4 among numerous swine barns, these sites provide ample opportunity for exposure to swine manure, and it is not surprising to observe higher abundances of this bacterium at these sites. Furthermore, the prevalence of this bacterium, which is higher than other sites strongly suggests that the detected antibiotics are likely derived from leakage of swine manure during agricultural activities.

Similarly, other species such as *Clostridium chartatabidum* and *Clostridium paraputrificum*, detected in the intestines of cattle [80] and swine [81,82], were also found to be more abundant in these sites 3 and 4. We also observed that *Turicibacter sanguinis*, found in the swine gastrointestinal tract [83] and in environments influenced by swine waste [84,85,86], showed a high prevalence at sites 3 and 4. The finding of these swine gut bacteria in the sediment suggests that swine manure may greatly affect the distribution and abundance of antibiotics in this sedimentary environment.

Interestingly, *Proteiniclasticum ruminis*, initially identified in yak rumen [87] and common in cattle manure and manure-based compost [88,89], was predominantly detected at site 3. This prevalence indicates that antibiotic contamination and the bacterial community at this site were affected not only by swine but also by cattle manure, underscoring the potential effects of various agricultural activities on antibiotic contamination at these sites.

Lastly, *Clostridium butyricum*, a butyrate-producing bacterium that is found in animal intestines [90,91], was identified as the most prevalent at site 5, adjacent to a chicken farm. The high prevalence of *C. butyricum* could be attributed to its use as a probiotic in poultry feed, aimed at enhancing intestinal health and growth performance [92,93,94]. Once administered, *C. butyricum* can be excreted into the environment through chicken manure, potentially altering the composition of bacterial communities in sediments. Consequently, our data suggest that the presence of antibiotics at site 5 could be related to the adjacent chicken farm.

Bacteria from other phyla, including Actinobacteria, Bacteroidetes, and Chloroflexi, have also been observed in these sedimentary environments. *Corynebacterium humireducens*, *Moheibacter stercoris*, and *Sphaerobacter thermophilus* have been detected in various environments associated with livestock activities, such as those involving manure [95,96] and compost [97,98,99]. The co-existence of these bacteria, along with those from the Firmicutes phylum, indicates a likelihood of manure contamination in these environments, thus suggesting a potential source of antibiotic contamination.

Our results suggest that animal gut bacteria could serve as indicators of livestock-related antibiotic contamination. Given that sites 3 and 4 are affected by a livestock manure treatment facility and numerous swine barns, respectively, it is plausible that the presence of antibiotics at these locations primarily originates from the effluent of animal wastewater. The high prevalence of *P. ruminis* at site 3 suggests that antibiotic contamination and the bacterial community at this site were affected not only by swine but also by cattle manure. Similarly, the high prevalence of *C. butyricum* at site 5 could be related to its use as a probiotic in poultry feed and its subsequent excretion into the environment through chicken manure.

However, verifying the relationship between livestock-related antibiotics and gut bacteria still poses limitations. For instance, *C. saudiense* has been isolated from the feces of rats [100,101] and humans [102,103,104]. In our study, *C. saudiense* was observed at site 2, but no antibiotics were detected in both surface water and sediment near the sampling site. Also, *T. sanguinis* and *M. stercoris* detected high abundance at sites 3 and 4, where the livestock-related area was highly impacted, which was also found in humans [105,106]. To overcome those limitations, more intense sampling points along with a larger study area should be conducted. The authors should discuss the results and how they can be interpreted from the perspective of previous studies and the working hypotheses. The findings and their implications should be discussed in the broadest context possible. Future research directions may also be highlighted.

## 5. Conclusions

This research investigated the differences in the bacterial community affected by varied antibiotic release sources in the stream. As the concentration of antibiotics increased, the richness and diversity of bacteria in the stream decreased. Considering the relationship between antibiotic source and bacterial community in the stream, the Clostridium genus included in the Firmicutes phylum was the most abundant bacterium in the sediment, indicating that these species could serve as indicators of livestock-sourced fecal contamination. In addition, the presence of livestock gut bacteria in the sediment along with the high concentration of antibiotics may suggest that fecal contamination from livestock activities could be a significant source of antibiotic contamination in the stream. This section is not mandatory but can be added to the manuscript if the discussion is unusually long or complex.

## Figures and Tables

**Table 1:** Chemical properties of surface water samples.

Location	pH ^1^	EC	TOC	T-N	T-P
	1:5	dS m^−1^	mg L^−1^	mg L^−1^	mg L^−1^
Site 1	7.53 ± 0.03 ^b^	0.45 ± 0.01 ^c^	12.11 ± 0.06 ^c^	16.90 ± 2.44 ^b^	0.44 ± 0.02 ^c^
Site 2	7.49 ± 0.01 ^b^	0.28 ± 0.03 ^c^	5.81 ± 0.08 ^d^	4.11 ± 0.52 ^c^	0.24 ± 0.03 ^c^
Site 3	6.98 ± 0.03 ^d^	18.69 ± 0.88 ^a^	265.96 ± 6.84 ^a^	289.43 ± 7.27 ^a^	9.77 ± 0.96 ^a^
Site 4	7.24 ± 0.21 ^c^	1.01 ± 0.11 ^c^	6.25 ± 0.07 ^d^	5.43 ± 0.24 ^c^	0.54 ± 0.06 ^c^
Site 5	7.87 ± 0.02 ^a^	4.15 ± 0.43 ^b^	98.02 ± 0.14 ^b^	5.80 ± 0.64 ^c^	3.87 ± 0.27 ^b^

^1^ All measurements were conducted in triplicate, with the data presented as the mean value accompanied by the standard deviation; different letters in rows indicate significant differences at *p* < 0.05.

**Table 2:** Physicochemical properties of sediment samples.

Location	Soil Texture	pH ^1^	EC	SOM	T-C	T-N
		1:5	dS m^−1^	%	g kg^−1^	g kg^−1^
Site 1	Loamy sand	6.94 ± 0.01 ^d^	0.44 ± 0.01 ^d^	1.41 ± 0.14 ^cd^	2.87 ± 0.99 ^d^	0.99 ± 0.37 ^d^
Site 2	Sandy loam	6.87 ± 0.01 ^e^	0.39 ± 0.01 ^d^	0.91 ± 0.15 ^d^	9.95 ± 2.07 ^c^	1.61 ± 0.26 ^c^
Site 3	Sandy loam	7.14 ± 0.03 ^c^	16.23 ± 0.13 ^a^	3.36 ± 0.12 ^a^	19.10 ± 1.29 ^a^	4.86 ± 0.16 ^a^
Site 4	Sandy loam	7.71 ± 0.05 ^a^	4.15 ± 0.04 ^b^	2.45 ± 0.57 ^b^	12.38 ± 1.40 ^c^	1.60 ± 0.11 ^c^
Site 5	Sandy loam	7.62 ± 0.01 ^b^	3.18 ± 0.14 ^c^	1.85 ± 0.20 ^c^	15.79 ± 0.85 ^b^	3.22 ± 0.14 ^b^

^1^ All measurements were conducted in triplicate, with the data presented as the mean value accompanied by the standard deviation; different letters in rows indicate significant differences at *p* < 0.05.

**Table 3:** Concentrations of detected antibiotics in surface water and sediment samples.

Samples	Location	Antibiotic Concentration ^1^ (Mean ± SD)						
		CTC ^2^	OTC	TC	TYL	SMZ ^3^	SMX	STZ
Surface water (μg L^−1^)	Site 1	0.17 ± 0.11 ^a^	0.20 ± 0.06 ^ab^	0.08 ± 0.01 ^b^	0.04 ± 0.01 ^b^	0.04 ± 0.02 ^a^	0.03 ± 0.01 *	ND ^4^
	Site 2	ND	ND	ND	ND	ND	ND	ND
	Site 3	0.11 ± 0.02 ^a^	0.31 ± 0.08 ^a^	0.14 ± 0.01 ^a^	0.01 ± 0.00 ^c^	ND	0.06 ± 0.01 *	0.07 ± 0.02
	Site 4	0.11 ± 0.01 ^a^	ND	0.13 ± 0.01 ^a^	0.25 ± 0.02 ^a^	0.02 ± 0.00 ^a^	ND	ND
	Site 5	ND	0.17 ± 0.01 ^b^	ND	0.01 ± 0.00 ^c^	0.02 ± 0.00 ^a^	ND	ND
Sediment (μg kg^−1^)	Site 1	ND	ND	ND	ND	3.70 ± 0.35 ^a^	ND	ND
	Site 2	ND	ND	ND	ND	ND	ND	ND
	Site 3	9.04 ± 0.15 ^†^	ND	7.62 ± 0.05	ND	4.27 ± 0.05 ^a^	ND	1.45 ± 0.05
	Site 4	8.86 ± 0.13 ^†^	ND	ND	5.22 ± 0.17	3.73 ± 0.08 ^a^	ND	ND
	Site 5	ND	ND	ND	ND	4.96 ± 2.22 ^a^	ND	ND

^1^ All measurements were conducted in triplicate, with the data presented as the mean value accompanied by the standard deviation; ampicillin, penicillin G, spiramycin, and sulfadiazine were not detected in any of the surface water and sediment samples examined; thus, they are excluded from this table. ^2^ Different letters in rows indicate significant differences at *p* < 0.05. ^3^ For samples detected at only two sites, *t*-tests were conducted at a 95% significance level; * significant at *p* < 0.01; ^†^ non significance. ^4^ ND: not detected.

**Table 4:** The number of OTUs and alpha diversity indices based on the 16S rRNA gene.

Sample	Number of Reads	OTUs	Good’s Coverage	Chao1	Shannon
Site 1	15,547	1646	94.6%	2237	9.16
Site 2	15,046	1476	96.0%	1903	8.85
Site 3	18,263	1621	97.3%	2148	8.91
Site 4	16,504	1579	96.3%	2138	8.51
Site 5	22,499	1741	95.8%	2285	9.07

**Table 5:** Prevalence and distribution of human and livestock gut bacteria in sediment samples.

Phylum	Species	Relative Abundance (%)					
		Site 1	Site 2	Site 3	Site 4	Site 5	Total
Actinobacteria	*Corynebacterium humireducens*	-	0.61	0.31	0.87	-	1.79
Bacteroidetes	*Moheibacter stercoris*	0.11	1.97	1.65	1.33	-	5.06
Chloroflexi	*Sphaerobacter thermophilus*	0.30	-	1.08	0.41	0.13	1.92
Firmicutes	*Clostridium butyricum*	0.26	0.52	2.06	1.81	3.16	7.81
	*Clostridium chartatabidum*	-	0.07	0.37	0.61	-	1.05
	*Clostridium paraputrificum*	-	-	0.21	0.75	-	0.96
	*Clostridium saudiense*	0.29	1.04	5.90	8.10	0.07	15.4
	*Proteiniclasticum ruminis*	0.24	0.49	4.34	0.64	1.22	6.93
	*Tissierella creatinophila*	-	-	0.40	-	-	0.4
	*Turicibacter sanguinis*	0.34	1.26	5.97	2.19	0.23	9.99
Total		1.54	5.96	22.29	16.71	4.81	51.31

## Data Availability

The data presented in this study are available on request from the corresponding author.

## Supplementary Materials

The following supporting information can be downloaded at: https://www.mdpi.com/article/10.3390/toxics12020135/s1, Figure S1: Flow diagram of analytical method for antibiotics in water and soil samples; Figure S2: Extracted ion chromatogram (XICs) of selected antibiotics from spiked (0.01 mg L^−1^) water samples; Figure S3: Extracted ion chromatogram (XICs) of selected antibiotics from spiked (0.01 mg L^−1^) soil samples; Table S1: LC-MS/MS analytical parameters for the analysis of antibiotics; Table S2: MS/MS parameters with multiple reaction monitoring (MRM) transitions for multi-residue analysis of selected antibiotics; Table S3: Linear regression equations and determination coefficients for selected antibiotics, as derived from standard solution and matrix-matched calibration curves; Table S4: Matrix effect (ME) evaluation for selected antibiotics through comparison the slopes of matrix-matched calibration curve and standard solution calibration curve; Table S5: Method detection limit (MDL) and limits of quantification (LOQ) for the determination of selected antibiotics in water and soil samples; Table S6: Recovery and precision of the developed LC-MS/MS method for simultaneous analysis of selected antibiotics in water and soil samples.
