# Supplementary material for: Difference of Microbial Community in the Stream Adjacent to the Mixed Antibiotic Effluent Source
Source: Toxics. 2024 Feb 7;12(2):135. doi: 10.3390/toxics12020135 (PMC10891948; doi:10.3390/toxics12020135)
Supplement: Supplementary file 1 [file toxics-12-00135-s001.zip › toxics-2805029-supplementary.pdf]

Table S1. LC-MS/MS analytical parameters for the analysis of antibiotics.

Instrument	Parameter	Conditions		
HPLC	Column	Zorbax Eclipse Plus C18 (3.5 μm, 4.6×150 mm)		
	Guard column	Security Guard Cartridge Kit		
	Column temperature	25℃		
	Mobile phase	A: 0.1% (v v^{-1}) formic acid in water<br>B: 0.1% (v v^{-1}) formic acid in acetonitrile		
	Flow rate	0.7 mL min^{-1}		
	Inject volume	5 μL		
	Gradient	Time	Mobile phase	
		(min)	A (%)	B (%)
		0	90	10
		2	90	10
		8	50	50
		10	100	0
		11	0	100
		11.1	90	10
		15	90	10
MS/MS	Mode	Electrospray ionization (ESI)		
	Polarity	Positive (+)		
	Sc.an type	Multiple reaction monitoring (MRM)		
	Curtain gas	30 psi		
	Collision gas	High		
	Ion source temperature	500 ℃		
	Ion spray voltage	5500 V		

Table S2. MS/MS parameters with multiple reaction monitoring (MRM) transitions for multi-residue analysis of selected antibiotics.

Class	Compound (Abbreviation)	Precursor Ion (m z⁻¹)	Product Ion (m z⁻¹)	DP[1)] (V)	EP[2)] (V)	CE[3)] (V)	CXP[4)] (V)
Internal standard	Simeton[5)]	198.1	**124.1**[6)]	71	10	27	6
			128.1			27	22
			100.1			39	18
Penicillins	Ampicillin (AMP)	349.9	**106.1**	6	10	41	14
			192.0			21	24
			113.9			41	8
	Penicillin G (PNG)	334.9	**217.0**	136	10	19	12
			202.0			31	24
			91.0			67	16
Tetracyclines	Chlortetracycline (CTC)	479.0	**444.0**	66	10	31	24
			462.1			23	12
			260.0			73	18
	Oxytetracycline (OTC)	461.1	**426.1**	36	10	27	18
			443.2			19	22
			201.1			49	12
	Tetracycline (TC)	445.1	**410.1**	26	10	29	20
			427.1			19	28
			154.2			37	14
Macrolides	Spiramycin (SPM)	843.5	**174.2**	101	10	27	8
			540.3			19	14
			101.0			23	10
	Tylosin (TYL)	916.5	**772.4**	61	10	45	4
			174.2			45	22
			83.1			129	2
Sulfonamides	Sulfadiazine (SDZ)	251.0	**156.0**	1	10	21	8
			92.0			33	12
			65.0			61	6
	Sulfamethoxazole (SMX)	279.0	**186.0**	1	10	23	10
			124.0			29	12
			65.0			67	8
	Sulfamethazine (SMZ)	254.0	**156.0**	1	10	21	14
			92.0			33	10
			65.0			61	6
	Sulfathiazole (STZ)	255.9	**155.9**	21	10	21	18
			92.0			33	12
			65.0			63	6

[1)] DP: decluttering potential, [2)] EP: entrance potential, [3)] CE: collision energy, [4)] CXP: collision cell exit potential, [5)] Simeton: internal standard, [6)] bold product ion was used for quantification.

Table S3. Linear regression equations and determination coefficients for selected antibiotics, as derived from standard solution and matrix-matched calibration curves.

Compound	Matrix	Linear Range (mg L^{-1} or mg kg^{-1})	Linear Regression Equation (y = ax + b)	Coefficient of Determination (R^2)
Ampicillin	Standard	0.01–1.0	y = 4.1677x − 0.0515	0.9998
	Water	0.01–1.0	y = 0.2678x + 0.0047	0.9956
	Soil	0.01–1.0	y = 0.3006x − 0.003	0.9973
Penicillin G	Standard	0.01–1.0	y = 0.2044x − 0.0034	0.9990
	Water	0.01–1.0	y = 0.0982x + 0.0022	0.9938
	Soil	0.01–1.0	y = 0.0397x + 0.0003	0.9998
Chlortetracycline	Standard	0.01–1.0	y = 4.3201x − 0.0569	0.9996
	Water	0.01–1.0	y = 0.6906x + 0.0109	0.9985
	Soil	0.01–1.0	y = 0.891x + 0.0065	0.9955
Oxytetracycline	Standard	0.01–1.0	y = 3.5338x − 0.0627	0.9992
	Water	0.01–1.0	y = 0.4321x + 0.007	0.9967
	Soil	0.01–1.0	y = 0.3147x + 0.0036	0.9978
Tetracycline	Standard	0.01–1.0	y = 6.9437x − 0.0933	0.9996
	Water	0.01–1.0	y = 2.0677x + 0.0272	0.9933
	Soil	0.01–1.0	y = 2.4183x + 0.0198	0.9977
Spiramycin	Standard	0.01–1.0	y = 1.9795x − 0.0257	0.9993
	Water	0.01–1.0	y = 0.9123x + 0.0223	0.9952
	Soil	0.01–1.0	y = 1.1597x + 0.0212	0.9916
Tylosin	Standard	0.01–1.0	y = 5.0753x − 0.4560	0.9998
	Water	0.01–1.0	y = 2.0855x + 0.0036	0.9988
	Soil	0.01–1.0	y = 1.8263x − 0.0073	0.9988
Sulfadiazine	Standard	0.01–1.0	y = 5.2131x – 0.0478	0.9995
	Water	0.01–1.0	y = 0.9011x + 0.012	0.9994
	Soil	0.01–1.0	y = 0.7949x − 0.005	0.9998
Sulfamethazine	Standard	0.01–1.0	y = 6.0890x − 0.0887	0.9995
	Water	0.01–1.0	y = 0.4277x −0.0016	0.9987
	Soil	0.01–1.0	y = 0.3962x + 0.003	0.9939
Sulfamethoxazole	Standard	0.01–1.0	y = 7.1538x −0.0493	0.9994
	Water	0.01–1.0	y = 0.6185x + 0.0182	0.9963
	Soil	0.01–1.0	y = 0.8815x + 0.0094	0.9989

Sulfathiazole	Standard	0.01–1.0	y = 1.2146x − 0.0084	0.9995
	Water	0.01–1.0	y = 0.6536x + 0.004	0.9995
	Soil	0.01–1.0	y = 0.8031x + 0.008	0.9971

Table S4. Matrix effect (ME) evaluation for selected antibiotics through comparison the slopes of matrix-matched calibration curve and standard solution calibration curve.

Compound	Matrix	Slope	Y-intercept	R^2	ME (%)
Ampicillin	Standard	4.E+06	−3.E+04	0.9942	-
	Water	4.E+06	2.E+05	0.9792	7.3
	Soil	3.E+06	1.E+05	0.9856	−11.1
Penicillin G	Standard	5.E+06	−9.E+04	0.9992	-
	Water	2.E+06	−6.E+04	0.9933	−52.4
	Soil	3.E+06	5.E+03	0.9953	−39.5
Chlortetracycline	Standard	2.E+07	−1.E+06	0.9799	-
	Water	4.E+07	2.E+05	0.9942	80.5
	Soil	3.E+07	−7.E+05	0.9979	24.2
Oxytetracycline	Standard	3.E+06	−2.E+05	0.9454	-
	Water	8.E+06	−3.E+05	0.9808	124.9
	Soil	1.E+07	−7.E+05	0.9519	198.9
Tetracycline	Standard	2.E+07	−1.E+06	0.9704	-
	Water	4.E+07	1.E+06	0.9980	101.5
	Soil	4.E+07	3.E+05	0.9855	81.7
Spiramycin	Standard	5.E+06	−6.E+04	0.9973	-
	Water	2.E+06	−1.E+04	0.9993	−54.2
	Soil	2.E+06	−1.E+05	0.8262	−64.2
Tylosin	Standard	5.E+07	−8.E+05	0.9993	-
	Water	3.E+07	1.E+06	0.9747	−35.8
	Soil	3.E+07	9.E+05	0.9927	−29.0
Sulfadiazine	Standard	1.E+07	−1.E+05	0.9998	-
	Water	7.E+06	3.E+05	0.9967	−42.8
	Soil	5.E+06	1.E+05	0.9814	−63.6
Sulfamethazine	Standard	3.E+07	−2.E+05	0.9919	-
	Water	2.E+07	−8.E+05	0.9457	−35.2
	Soil	8.E+06	8.E+04	0.9422	−68.1
Sulfamethoxazole	Standard	2.E+07	5.E+05	0.9776	-
	Water	6.E+06	−1.E+03	0.9968	−62.9
	Soil	5.E+06	3.E+05	0.9300	−72.7
Sulfathiazole	Standard	1.E+07	2.E+05	0.9841	-

Water	6.E+06	−2.E+05	0.9826	−52.7
Soil	4.E+06	4.E+04	0.9274	−70.3

Table S5. Method detection limit (MDL) and limits of quantification (LOQ) for the determination of selected antibiotics in water and soil samples.

Class<br>Compound	Water		Soil	
	MDL ($ng\ L^{-1}$)	LOQ ($ng\ L^{-1}$)	MDL ($ng\ kg^{-1}$)	LOQ ($ng\ kg^{-1}$)
Penicillins				
Ampicillin	10.9	34.5	13.2	42.0
Penicillin G	12.0	38.1	8.5	27.0
Tetracyclines				
Chlortetracycline	7.7	24.5	2.9	9.3
Oxytetracycline	8.7	27.6	8.7	27.6
Tetracycline	12.3	39.2	8.2	26.3
Macrolides				
Spiramycin	2.2	7.0	1.2	4.0
Tylosin	2.1	6.6	4.9	15.6
Sulfonamides				
Sulfadiazine	8.8	28.1	5.9	18.9
Sulfamethazine	6.6	20.9	2.9	9.2
Sulfamethoxazole	6.1	19.3	11.9	37.8
Sulfathiazole	7.2	22.8	3.6	11.3

Table S6. Recovery and precision of the developed LC-MS/MS method for simultaneous analysis of selected antibiotics in water and soil samples.

Class<br>Compound	Concentration (mg L^{-1})	Water		Soil	
		Recovery (%)	RSD (%)	Recovery (%)	RSD (%)
Penicillins					
Ampicillin	0.1	97.7	10.9	72.5	2.6
	1.0	82.1	1.7	62.4	9.3
Penicillin G	0.1	91.7	12.2	79.8	10.5
	1.0	101.6	0.9	78.9	9.1
Tetracyclines					
Chlortetracycline	0.1	74.6	5.1	72.9	9.9
	1.0	87.4	2.7	90.0	1.6
Oxytetracycline	0.1	82.9	2.8	86.4	7.2
	1.0	92.2	6.6	110.0	12.7
Tetracycline	0.1	114.5	0.7	99.9	4.0
	1.0	113.0	2.1	111.6	2.9
Macrolides					
Spiramycin	0.1	36.8	5.5	50.9	1.0
	1.0	59.1	6.7	57.5	2.4
Tylosin	0.1	103.2	5.8	96.6	5.4
	1.0	115.8	3.9	99.5	2.2
Sulfonamides					
Sulfadiazine	0.1	112.8	7.7	79.0	9.5
	1.0	85.8	11.6	77.2	9.9
Sulfamethazine	0.1	80.7	3.1	77.4	8.1
	1.0	112.4	5.0	74.3	11.1
Sulfamethoxazole	0.1	93.0	6.1	62.8	7.8
	1.0	102.1	2.0	88.7	5.5
Sulfathiazole	0.1	61.9	4.7	60.4	7.5
	1.0	63.3	1.3	65.9	7.0

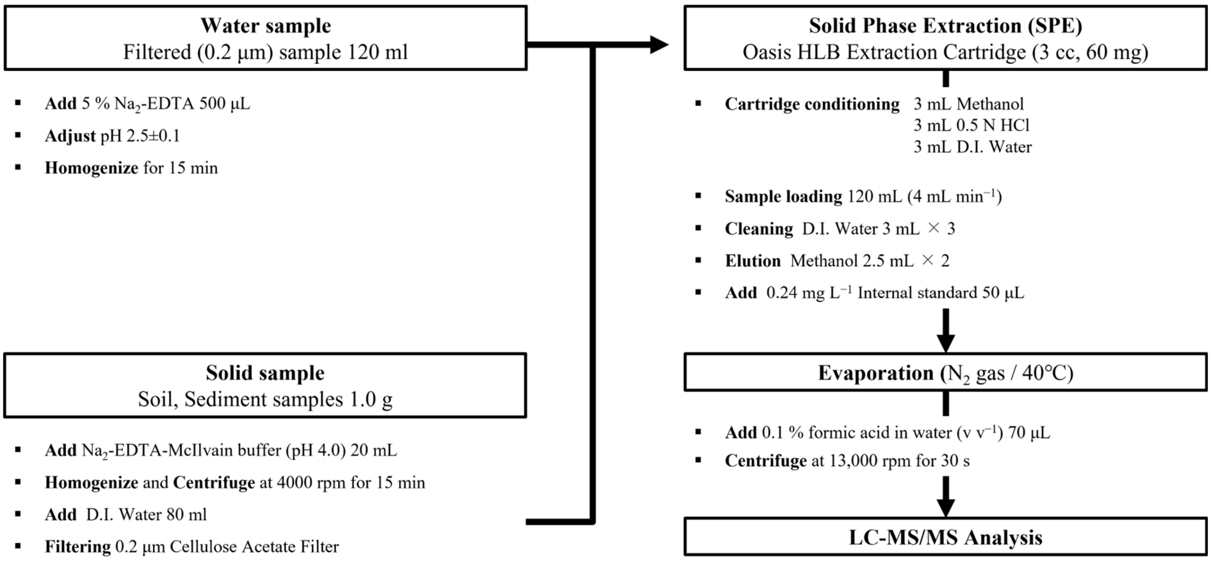

Figure S1. Flow diagram of analytical method for antibiotics in water and soil samples.

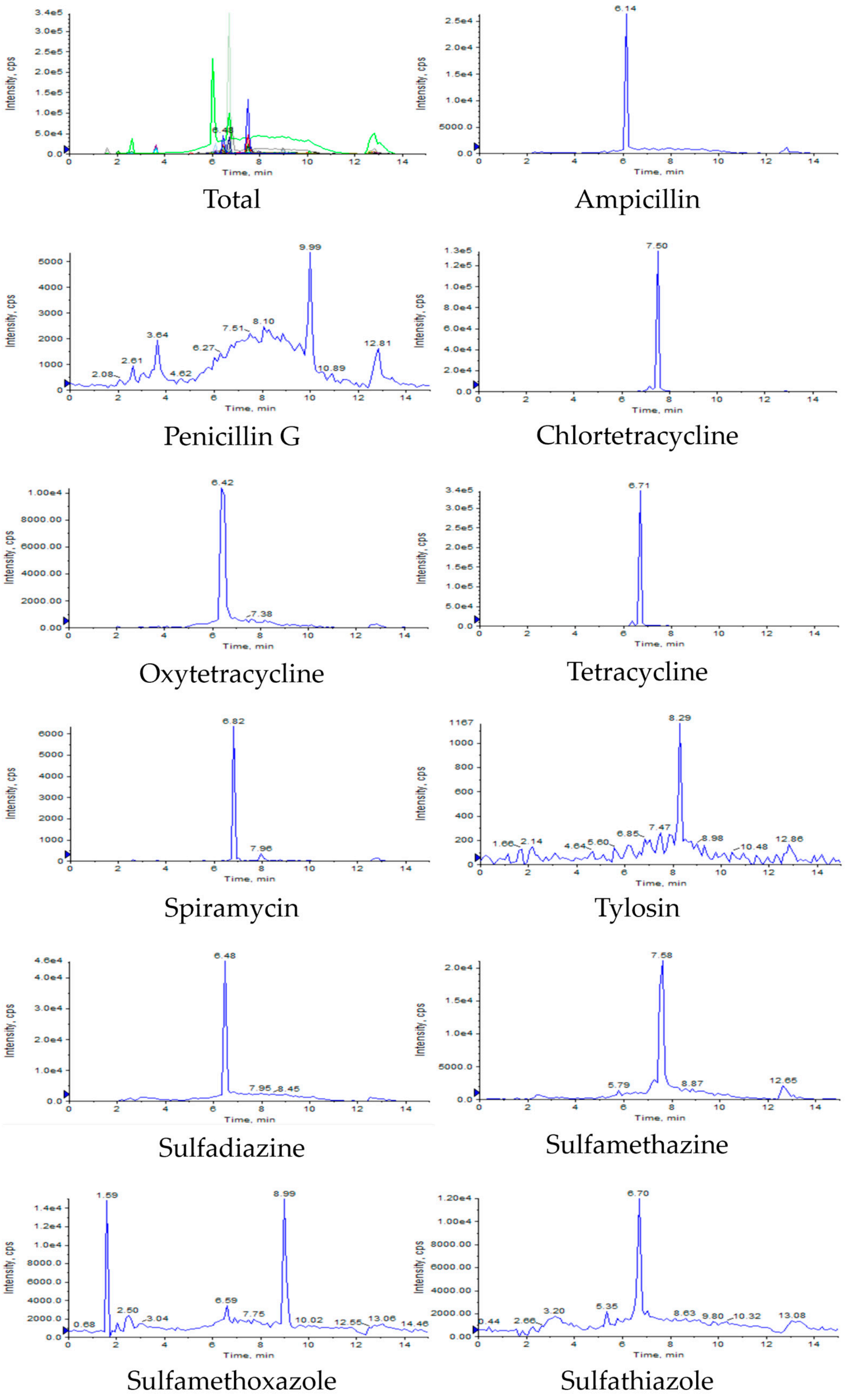

Figure S2. Extracted ion chromatogram (XICs) of selected antibiotics from spiked (0.01 mg L^{-1}) water samples.

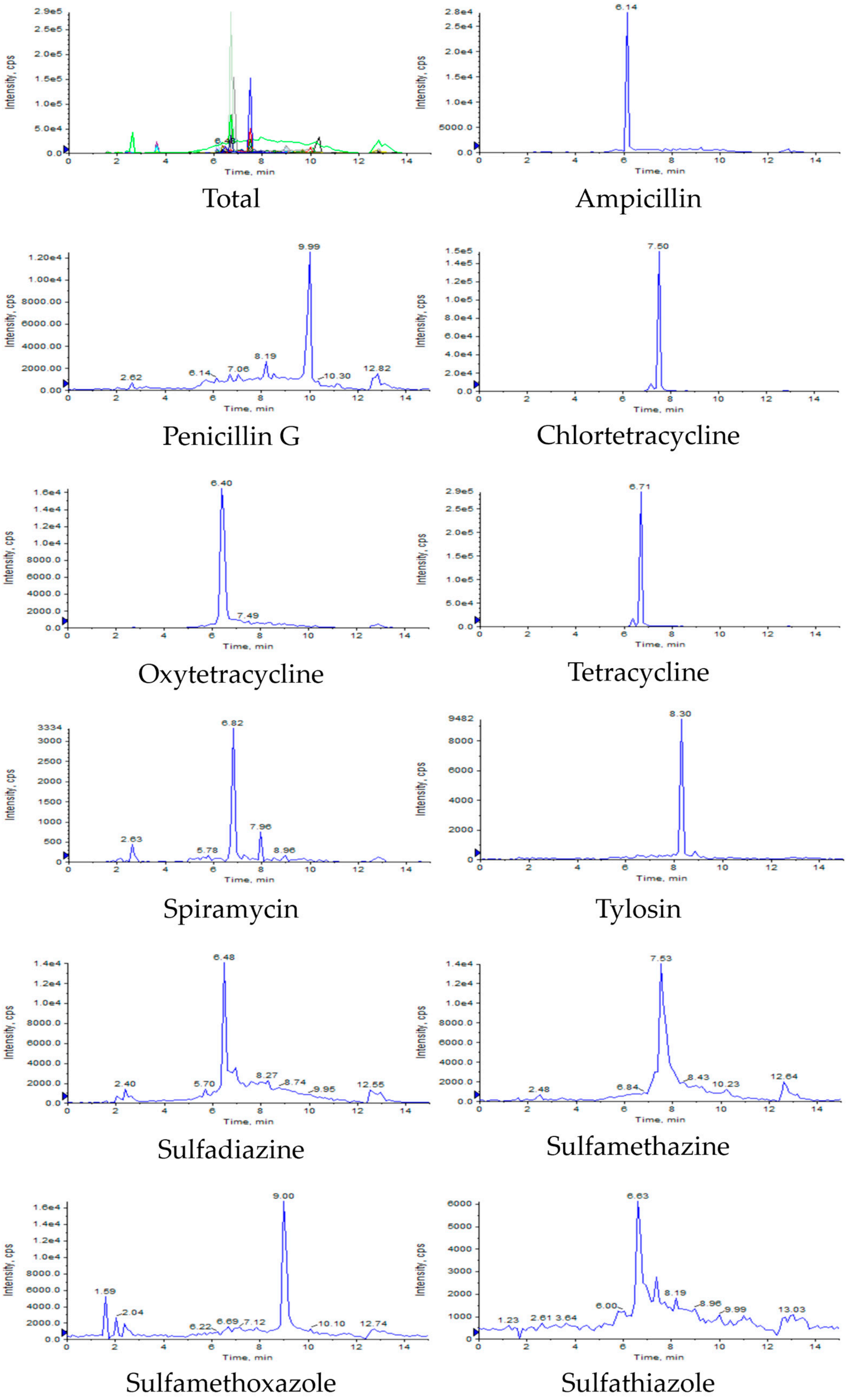

Figure S3. Extracted ion chromatogram (XICs) of selected antibiotics from spiked (0.01 mg kg^{-1}) soil samples.
